# Dynamic Mechanical Properties and Thermal Properties of Longitudinal Basalt/Woven Glass Fiber Reinforced Unsaturated Polyester Hybrid Composites

**DOI:** 10.3390/polym13193343

**Published:** 2021-09-29

**Authors:** Nur Izzah Nabilah Haris, R. A. Ilyas, Mohamad Zaki Hassan, S. M. Sapuan, Atiqah Afdzaluddin, Khairur Rijal Jamaludin, Sheikh Ahmad Zaki, Faizir Ramlie

**Affiliations:** 1 Institute of Advanced Technology, Universiti Putra Malaysia, Serdang 43400, Malaysia; nurizzahnabilah.haris@gmail.com; 2 School of Chemical and Energy Engineering, Faculty of Engineering, Universiti Teknologi Malaysia, Johor Bahru 81310, Malaysia; ahmadilyas@utm.my; 3 Centre for Advanced Composite Materials (CACM), Universiti Teknologi Malaysia, Johor Bahru 81310, Malaysia; 4 Razak Faculty of Technology and Informatics, Universiti Teknologi Malaysia, Jalan Sultan Yahya Petra, Kuala Lumpur 54100, Malaysia; khairur.kl@utm.my (K.R.J.); faizir.kl@utm.my (F.R.); 5 Laboratory of Biocomposite Technology, Institute of Tropical Forestry and Forest Products (INTROP), Universiti Putra Malaysia, Serdang 43400, Malaysia; sapuan@upm.edu.my; 6 Advanced Engineering Materials and Composites Research Centre (AEMC), Department of Mechanical and Manufacturing Engineering, Faculty of Engineering, Universiti Putra Malaysia, Serdang 43400, Malaysia; 7 Institute of Microengineering and Nanoelectronics, Universiti Kebangsaan Malaysia, Bangi 43600, Malaysia; a.atiqah@ukm.edu.my; 8 Malaysia-Japan International Institute of Technology, Universiti Teknologi Malaysia, Jalan Sultan Yahya Petra, Kuala Lumpur 54100, Malaysia; sheikh.kl@utm.my

**Keywords:** hybrid composite, glass fiber, basalt fiber, DMA, TMA

## Abstract

This study investigates the mechanical, thermal, and chemical properties of basalt/woven glass fiber reinforced polymer (BGRP) hybrid polyester composites. The Fourier transform infrared spectroscopy (FTIR) was used to explore the chemical aspect, whereas the dynamic mechanical analysis (DMA) and thermomechanical analysis (TMA) were performed to determine the mechanical and thermal properties. The dynamic mechanical properties were evaluated in terms of the storage modulus, loss modulus, and damping factor. The FTIR results showed that incorporating single and hybrid fibers in the matrix did not change the chemical properties. The DMA findings revealed that the B7.5/G22.5 composite with 7.5 wt% of basalt fiber (B) and 22.5 wt% of glass fiber (G) exhibited the highest elastic and viscous properties, as it exhibited the higher storage modulus (8.04 × 10^9^ MPa) and loss modulus (1.32 × 10^9^ MPa) compared to the other samples. All the reinforced composites had better damping behavior than the neat matrix, but no further enhancement was obtained upon hybridization. The analysis also revealed that the B22.5/G7.5 composite with 22.5 wt% of basalt fiber and 7.5 wt% of glass fiber had the highest T_g_ at 70.80 °C, and increased by 15 °C compared to the neat matrix. TMA data suggested that the reinforced composites had relatively low dimensional stabilities than the neat matrix, particularly between 50 to 80 °C. Overall, the hybridization of basalt and glass fibers in unsaturated polyester formed composites with higher mechanical and thermal properties than single reinforced composites.

## 1. Introduction

Fiber-reinforced polymer (FRP) composites are fabricated by blending reinforcing fibers with a polymer matrix [1]. In nearly all cases, combining a matrix and reinforcing fiber results in composites with higher mechanical properties compared to the single components [2,3]. Optimization of the fabrication parameters are often used to achieve an optimum level of overall composite quality [4,5,6]. However, under certain circumstances, the properties of single FRP composites might fail to meet the desired level of characteristics. Accordingly, scientists began to incorporate more than one type of reinforcing fibers in a single matrix, forming hybrid FRP composites [7,8,9]. The synergistic effects of hybridization between more than one fiber could overcome the drawbacks of single FRP composites. This technique is quickly gaining in popularity due to its ability to give the flexibility to alter the composite behavior, which is not possible with single FRB composites. It is essential to note that if the hybrid arrangement is not carefully planned, the resulting hybrid composite may have a lesser strength than its parts.

Glass fiber is a fine synthetic fiber made up of glass that typically comprises more than 50% of silica along with various mineral oxides such as calcium, iron, and aluminum oxides. It is extensively used as thermal, acoustical, and electrical insulation on its own [10]. Moreover, it is utilized as a reinforcing material for polymer composites owing to its lightweight characteristic, strength, versatility, and robustness [11]. Glass fiber reinforced composites are good in terms of performance and cost. Due to its reasonable price and unique properties, it is one of the most prominent reinforcing agents in the current and future segments [12]. Basalt fiber is a mineral-sourced fiber that originates from volcanic magma. The basalt fiber is manufactured by crushing, melting, and extruding molten basalt rocks through small nozzles, producing a fibrous form of basalt. It offers high thermal and chemical resistance, good adhesion to polymer, and high elastic modulus [13]. 

Recently, several experiments have been conducted to investigate the mechanical characteristics of the basalt/glass fiber reinforced polymer (BGRP), as shown in Table 1. 

Basalt fiber is one of the materials considered as reinforcement with polymer composite. The incorporation of this fiber within the polymer composite, has resulted in high operating temperature range, excellent heat, good strength, good chemical resistance, low water absorption, and good sound insulation properties [14,15,16,17,18,19]. Barczewski et al. [20] evaluated the effect of hybridizing mineral basalt (B) powder with glass fiber in the polypropylene (PP) matrix using injection molding. It was discovered that increasing the amount of B filler resulted in decreasing the mechanical properties of the composites. A similar finding was also reported by Mahesh Babu et al. [21], whereby an increase in B filler caused a lower impact strength. Furthermore, the influence of the stacking sequence of hybrid BGRP laminates in terms of mechanical properties was also observed by El-Wazery [22]. It was revealed that there was an enhancement in the mechanical properties of basalt/carbon-reinforced polymer (BCRP) composites with stacking sequences (Carbon/Basalt/Carbon/Basalt/Carbon) as compared to the other stacking sequences (Glass/Basalt/Glass/Basalt/Glass). Moreover, the layering arrangement such as (Glass/Glass/Basalt/Glass/Basalt/Basalt) offered an outstanding tensile performance than the other laminate configurations. However, the hybrid composite still portrayed a lower performance as compared to the plain BFRP and GFRP composites [23]. Most of the studies reported that lamination between the basalt and glass fibers could offer a lower mechanical property as compared with the plain basalt or glass composite laminates [24,25,26]. Bozkurt et al. [24] evaluated the influence of the woven basalt fiber with an E-glass fabric reinforced with epoxy resin using vacuum-assisted resin transfer molding. By increasing the basalt fiber content in all the hybrid composite samples, considerable deteriorations in tensile strength and modulus were observed. In addition, Abd El-Baky et al. [25] mentioned that the tensile characteristics of BGRP composites were inferior compared to GFRP laminates. The authors advised that increasing the G-fiber content enhances the mechanical behavior. Likewise, Patel et al. [24] had concluded that the hybrid BGRP laminate showed the highest tensile strength. However, it demonstrated a lower impact strength as compared to the neat glass laminate. Interestingly, Jain et al. [27] developed hybrid BGRP composites with better flexural strength and lower tensile strength than the BRFP laminate. It runs contrary to the observations reported by Abd El-Baky et al. [25]. Moreover, Ozbek et al. [26] reported a lower value of impact strength of the BGRP sample. However, the result was comparably low in comparison to the levels described in the literature. In contrast, Sapuan et al. [28] stated that the findings obtained in terms of the mechanical characteristics surpass the previous works in this field. The BGRP samples significantly offered high tensile properties as well as flexural and impact strength values at a composition of 22.5 wt% of G and 7.55 wt% of B fabrics.

Despite the fact that numerous studies have attempted to fabricate BGRP hybrid composites, only a few or no extensive investigation was reported on the mechanical, thermal, and chemical properties of BGRP hybrid unsaturated polyester (UP) composite. On that account, this study was performed to fabricate hybrid unsaturated polyester composites at various compositions of reinforcing fibers using the hand layup technique. The mechanical and thermal properties of fabricated composites were analyzed using DMA and TMA.

## 2. Materials and Methods

### 2.1. Materials

The hybrid composites comprise roving basalt fiber, woven E-glass fiber, unsaturated polyester resin matrix, and methyl ethyl ketone peroxide (MEKP) catalyst. The roving basalt fiber was purchased from Basaltex NV (Wevelgem, Belgium), whereas the woven E-glass fiber was provided by Innovative Pultrusion Sdn. Bhd. (Negeri Sembilan, Malaysia). The chemical constituents and physical properties of the fibers are listed in Table 2 and Table 3, respectively.

### 2.2. Fabrication of B/G/UP Hybrid Composites

**Figure 1:**
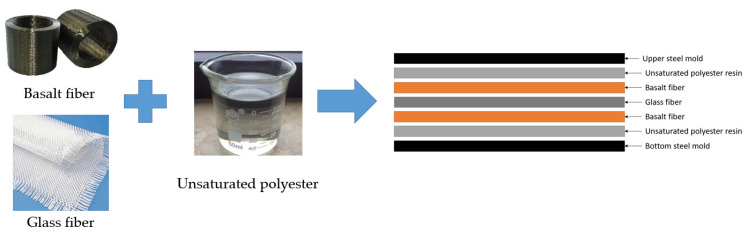
Typical lamination process of basalt fiber and glass fiber plies.

Initially, the basalt fiber was cut into 300 mm pieces, while the woven glass fiber was cut into 300 × 300 mm squares to fit into a 300 × 300 × 5 mm steel mold. Individual layers of fiber were manually laid in the mold and compressed at a compression load of 40 tons. Figure 1 shows the method of laminating basalt fiber with glass fiber plies.

The unsaturated polyester resin and the catalyst were combined with compressed fiber, yielding sandwich-structured hybrid composites. The compositions of fabricated composites are depicted in Table 4.

The numbers in the sample designation indicate the composition of basalt and glass fibers. One neat unsaturated polyester resin and two non-hybrid reinforced composites were also fabricated. The matrix to fiber ratios for all the hybrid and single reinforced composites were maintained at 7:3. An example calculation of hybrid composite (sample B15/G15) is tabulated in Table 5.

### 2.3. Chemical Structure of Composites 

The FTIR spectra of B/GF/UP composites were obtained using an IR spectrometer (Thermo Nicolet Corporation, Nicolet 6700 AEM, Madison, WI, USA) in the range of 4000 to 400 cm^−1^ at a 4 cm^−1^ resolution. The composite samples were mixed with KBr, compacted into pellets, and analyzed afterwards. 

### 2.4. Dynamic Mechanical Analysis

A dynamic mechanical analyzer (TA Instruments, Q800 DMA, New Castle, DE, USA) was used to determine the storage modulus, loss modulus, and damping factor of B/GF/UP composites as a function of temperature (−100 to 150 °C). The frequency and heating rate were fixed at 1 Hz and 10 °C·min^−1^, respectively. 

### 2.5. Thermomechanical Analysis

The dimensional changes of B/GF/UP composites as a function of temperature were measured using a thermomechanical analyzer (TA Instruments, TMA Q400, New Castle, DE, USA) per ASTM E831 [30]. The analysis was performed under a constant flow of nitrogen gas within a temperature range of −100 to 150 °C at a 5 °C·min^−1^ heating rate.

## 3. Results and Discussion

### 3.1. Chemical Structure of Composites 

**Figure 2:**
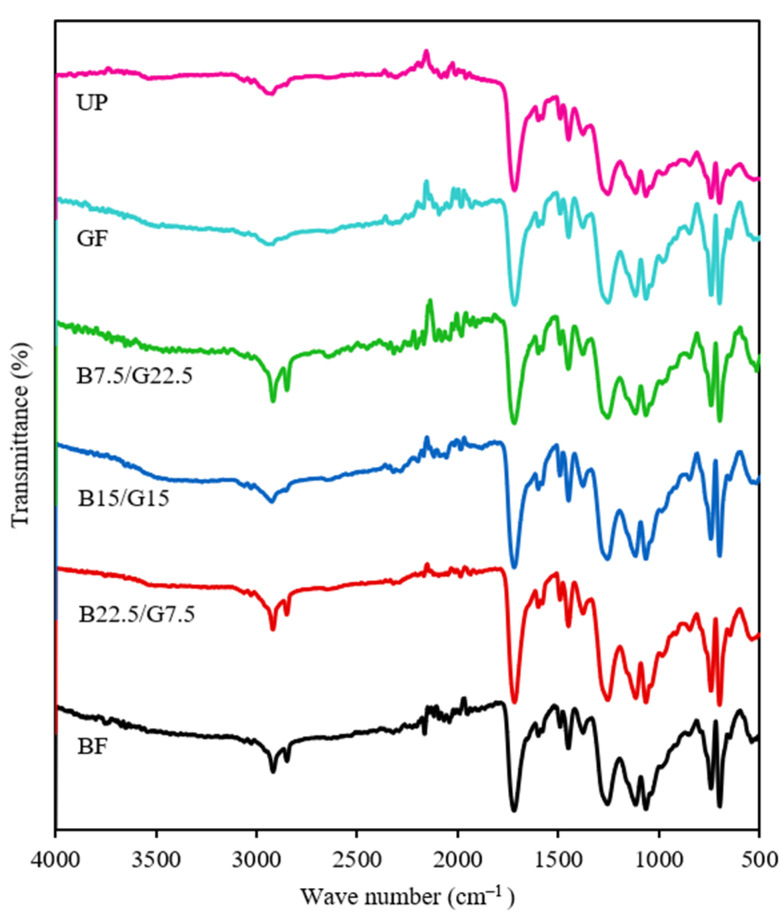
FTIR spectra of BF, B22.5/G7.5, B15/G15, B7.5/G22.5, GF, and UP composites.

The FTIR spectra for the neat polyester and basalt/woven glass fiber reinforced composites from 4000 to 400 cm^−1^ wavenumbers are shown in Figure 2. Based on the UP spectrum, some low intensity peaks appeared at high-range wavenumbers (>2000 cm^−1^) at 3530 cm^−1^ (O–H stretching), 2920 cm^−1^, and 2850 cm^−1^ (C–H stretching) [31]. At lower wavenumbers, numerous intense bands were observed at 1715 cm^−1^ (ester C=O stretching), 1600 cm^−1^ (ester C=O stretching), 1462 cm^−1^, 1450 cm^−1^, (C–H bending), 1250 cm^−1^ (aromatic ester C–O stretching), 1066 cm^−1^ (C–O stretching), and 703 cm^−1^ (C–H bending) [31,32,33]. All the identified bands are attributed to the chemical moieties in the unsaturated polyester chemical structure. Similar neat UP spectra were also reported by Arrieta et al., Chukwu et al., and Koto and Soegijono [31,32,33]. 

Upon incorporating fiber reinforcements, slight changes in the composites’ chemical characteristics were observed. For the single glass fiber reinforced composite (GF), the intensity of all the absorbance peaks appeared to be lower than the neat UP, but no new peak was identified. This observation suggests that the addition of glass fiber did not modify the chemical properties of the composite. As no functional groups are present on the glass fiber’s surface, no chemical modification can occur unless the glass fiber is chemically modified or grafted prior to incorporation in the polymer matrix [34]. Regarding the single basalt fiber reinforced composite (BF), the only apparent difference in the spectrum is the intensified peak at 2920 and 2850 cm^−1^ (C–H stretching). It was previously reported that the basalt fiber would exhibit a single absorbance peak at 1000 to 1200 cm^−1^ due to the Si-O-Si bond vibration and a minor peak at 2800 to 2900 cm^−1^ due to C–H stretching, which explains the intensified peak for the C–H stretching bands [35,36]. 

With regards to the hybrid fiber reinforced composites FTIR spectra (B22.5/G7.5, B15/G15, and B7.5/G22.5), no apparent differences between the single and hybrid composites were observed. All the characteristic bands that were observed in the spectra also appeared in the neat UP matrix. On this basis, it can be inferred that the hybridization of basalt and glass fibers in the matrix does not alter the chemical properties of the composites. Several studies have proposed to chemically treat the basalt fiber to improve the chemical bonding and fiber/matrix adhesion. In particular, Seghini et al. [36] conducted plasma polymerization on basalt fiber to enhance the adhesion with an epoxy matrix and found some chemical changes on the fiber surface via FTIR. 

### 3.2. Dynamic Mechanical Properties

#### 3.2.1. Storage Modulus

**Figure 3:**
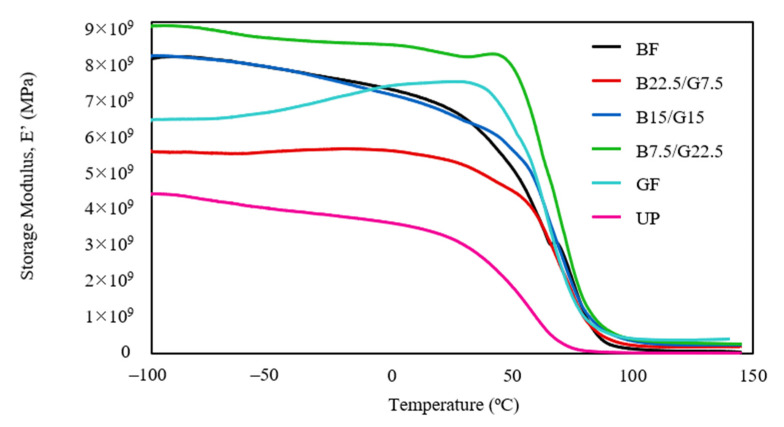
Storage modulus (E’) of BF, B22.5/G7.5, B15/G15, B7.5/G22.5, GF, and UP composites.

The storage modulus (E’) is a measure of the stored energy in the elastic structure of a material that corresponds to the material’s elastic response. This variable is particularly beneficial to evaluate the stiffness and elastic behavior of composites. The variation of the storage modulus with respect to the temperature for the fabricated composites are illustrated in Figure 3. 

As evident from the storage modulus plot, the single reinforced composites (BF and GF) had higher storage modulus values compared to the neat UP. In particular, the GF composite showed a more substantial increment of storage modulus, from 3.70 × 10^9^ MPa (UP) to 7.37 × 10^9^ MPa at a near transition temperature range within 0 to –50 °C. Upon hybridization of both fibers, only the B7.5/G22.5 hybrid composite had a higher storage modulus (8.04 × 10^9^ MPa) than BF (6.40 × 10^9^ MPa) and GF (7.37 × 10^9^ MPa). It appeared that the hybridization composition in B22.5/G7.5 and B15/G15 had a negative effect on the composites’ elasticity as revealed by the reduction of storage modulus as compared to BF and GF. The high storage modulus of B7.5/G22.5 (high proportion of glass fiber) indicates that the glass fiber primarily contributes to the enhanced elastic property of the hybrid composite. This statement is supported by the higher storage modulus GF than the BF composite, as shown in Figure 2. It can be deduced that a high proportion of glass fiber and a small amount of basalt in the hybrid composite promotes an efficient stress transfer between the reinforcing fibers and the unsaturated polyester composite, creating a positive hybrid effect on the elasticity [37,38]. A similar observation was also reported by Barczewski et al. for the glass fiber/basalt powder polypropylene hybrid composite, whereby a hybrid composite with higher glass fiber content portrayed the highest storage modulus [20]. The presence of basalt powder in the composite caused a gradual reduction of elasticity. The current finding also accords with the elastic modulus reported in their work. At a temperature range beyond 90 °C, the storage modulus values of all the composites dropped to a minimum and remained plateau, which portrays that the matrix has reached a rubbery state [39]. 

#### 3.2.2. Loss Modulus

**Figure 4:**
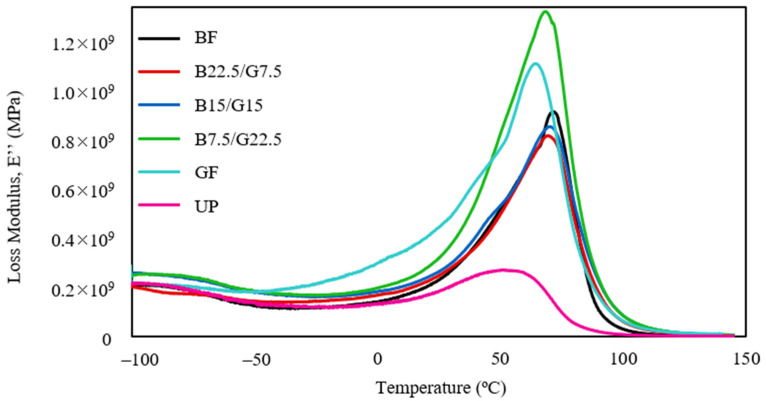
Loss modulus (E”) of BF, B22.5/G7.5, B15/G15, B7.5/G22.5, GF, and UP composites.

Contrary to the storage modulus, the loss modulus (E”) is helpful to evaluate the viscous attribute of a polymeric material [40]. The quantification of the parameter is made based on the energy lost, that is dissipated as heat, when subjected to a load cycle [41]. The loss modulus plots of single and hybrid reinforced unsaturated polyester composites within −100 to 150 °C are depicted in Figure 4.

Apparently, all the composites exhibited similar loss modulus curve trends. A gradual increase at the low-temperature range (<50 °C), a linear surge at the transition region (50–70 °C), and eventually a steep drop of loss modulus at higher temperatures. The highest point of the loss modulus curve can be used to compute the glass transition temperature (T_g_). The neat UP showed the lowest loss modulus peak at 2.74 × 10^8^ MPa, implying that the polyester molecular chains are more mobile without the fiber reinforcement [42]. All the reinforced composites exhibited remarkably high loss modulus particularly B7.5/G22.5 and GF composites with 1.32 × 10^9^ MPa and 1.10 × 10^9^ MPa peak modulus values. The higher peak values suggest that both composites, especially B7.5/G22.5, have a good fiber-matrix bond strength compared to the rest. Both composites comprised higher loadings of glass fiber than the other composites, indicating that the glass fiber reinforcement is capable of improving the viscous property. The hybridization of basalt at 7.5 wt% and glass fiber at 22.5 wt% seemed to have a notable enhancement effect on the loss modulus. The enhancement of glass fiber reinforced composites’ viscous response for the sisal/woven glass fiber reinforced polyester composite with higher glass fiber loading was also reported by Gupta and Deep [43]. The B22.5/G7.5 and B15/G15 hybrid composites showed marginally lower loss modulus peaks than the BF composite. Although exhibiting no viscous response improvement, the addition of basalt fiber at higher composition in the matrix had improved the composites’ thermal stability, as evidenced by the peak shifting to a higher temperature range. This thermal stability enhancement is ascribable to the polymer chains segmental motion restriction caused by the reinforcing fiber [44]. The T_g_ derived from the loss modulus curve will be described shortly. 

#### 3.2.3. Tan δ

**Figure 5:**
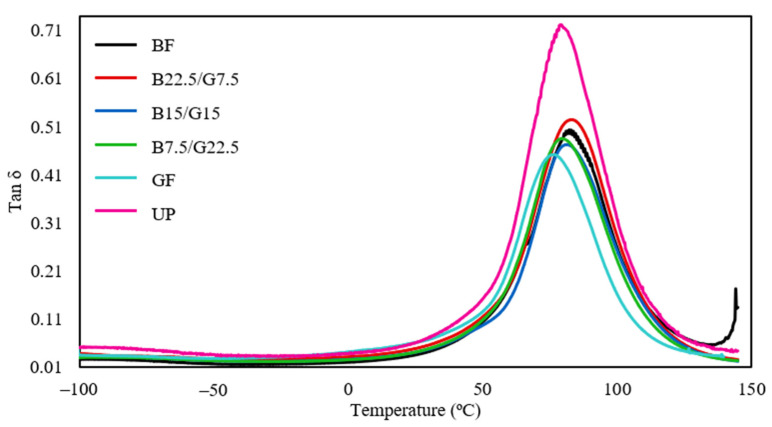
Tan δ of BF, B22.5/G7.5, B15/G15, B7.5/G22.5, GF, and UP composites.

The tan δ or damping factor is the ratio of loss modulus to storage modulus that represents a material’s impact resistance and its elastic and viscous phase weightage [39,45]. A high tan δ value indicates that the material is more viscous and has high energy dissipation potential, whereas a low tan δ is associated with a highly elastic material capable of storing energy within its structure [46]. Figure 5 displays the tan δ plots of the fabricated single and hybrid reinforced unsaturated polyester composites within −100 to 150 °C. The peak tan δ values along with the T_g_ derived from the tan δ and loss modulus curves are summarized in Table 6. 

As evident in the plot, UP showed the highest tan δ peak, indicating a more viscous characteristic with high polymeric chain mobility. Single and hybrid fiber reinforcements in the matrix have substantially affected the damping property of the composites, making them more elastic than the neat matrix as observable by the lowered peaks of tan δ. Among all the reinforced composites, the GF composite showed the most significant tan δ reduction relative to the UP composite by 38% from 0.71 to 0.44. The BF composite portrayed a slightly lower tan δ peak reduction, which was 32% compared to the neat matrix. The incorporation of reinforcing fibers has led to restricted polymeric chain mobility due to strong adhesion at the fiber-matrix interface, and thus lowers the flexibility of the composite [46]. All the hybrid composites, namely B22.5/G7.5, B15/G15, and B7.5/G22.5 exhibited roughly similar reductions of tan δ. Based on this observation, it is inferred that the hybrid reinforcement has a negligible effect on the damping property of the composite. 

With respect to the T_g_ derived from the tan δ plot, it appeared that the incorporation of single basalt fiber had marginally improved the thermal stability as evidenced by the increase of T_g_ from 80.24 to 81.37 °C. However, the inclusion of single glass fiber reinforcement had reduced the T_g_ from 80.24 to 76.98 °C. Higher T_g_ values were obtained for hybrid composites with higher loadings of basalt fiber relative to glass fiber. This finding suggests that basalt fiber is more prominent than glass fiber when it comes to enhancing the thermal stability of the composites. The higher thermal stability indicates that the composite has better molecular chain crosslinking in its structure [47]. A similar trend was found based on the T_g_ values derived from loss modulus peaks. The hybridization of both fibers had resulted in composites with intermediate T_g_ values within 70.80 and 69.70 °C, indicating a moderate improvement of interface bonding between the matrix and fiber [48]. The inclusion of basalt and glass fibers had decreased the mobility of the polymer chain, yielding higher T_g_ for single and hybrid composites than the UP specimen. Amuthakkannan and Manikandan reported a similar T_g_ of basalt fiber reinforced UP composite at approximately 68 °C [49]. The T_g_ of glass fiber reinforced UP composite from this study also accords with those reported by Subrata et al. at 71.35 °C [50]. Comparing the T_g_ values derived from both methods, the trend exhibited by the data of loss modulus is more realistic, whereby all the reinforced fiber showed improved thermal stability. In particular, single reinforced composites showed the highest and lowest increment, whereas the hybrid composites showed an intermediate improvement of thermal stability. The validity and more realistic data of T_g_ obtained from the loss modulus was also reported by numerous researchers that dealt with hybrid reinforced composites [42,43]. 

### 3.3. Thermomechanical Properties

**Figure 6:**
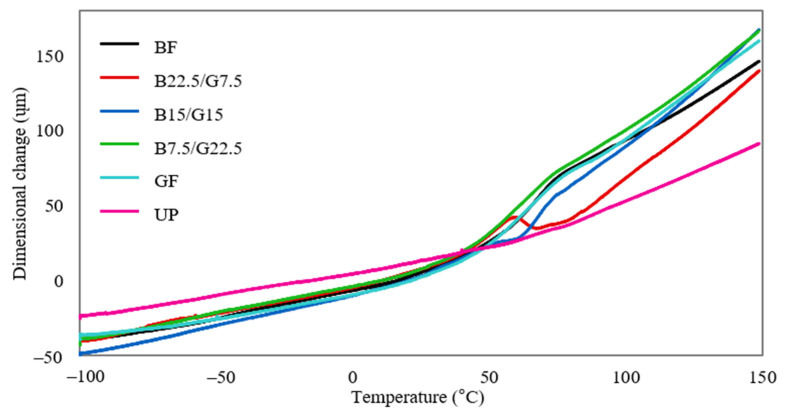
Thermomechanical analysis plots of BF, B22.5/G7.5, B15/G15, B7.5/G22.5, GF, and UP composites.

TMA is a straightforward and convenient tool to evaluate the thermal properties of a polymeric material. The instrument measures and records the changes of a sample’s dimension as it is subjected to heat or load, revealing information on the structure, composition, and potential application of the material [51]. The TMA result of the fabricated single and hybrid basalt/woven glass fiber reinforced unsaturated polyester composites is presented in Figure 6.

Based on the plot, it is evident that the UP composite exhibited the lowest extent of expansion and shrinkage among all the examined composites. This finding suggests that the neat matrix has better dimensional stability when subjected to temperature variation [52]. The reinforced composites portrayed a more notable expansion and shrinkage upon temperature changes, implying that fiber reinforcement at all fiber loadings caused an undesired effect on the dimensional properties. The detrimental effect is more obvious between 50 to 80 °C than the other temperatures, whereby all the reinforced composites exhibited steep increments of dimension. BF, B15/G15, B7.5/G22.5, and GF showed a similar behavior of dimensional change as evidenced by the overlapping curves in the TMA plot. As the temperature gradually increased, the dimension increased correspondingly, implying the occurrence of faster and unrestrained molecular movements and lack of fiber/matrix interaction [53]. On the other hand, B22.5/G7.5 had a slightly different response, whereby a sudden shrinkage occurred from 60 to 67 °C. The shrinkage indicates the penetration of reinforcing fibers into the polymer matrix [54]. Asim et al. [54] reported a similar behavior of dimensional change for the pineapple leaf/kenaf fiber reinforced phenolic composite. Overall, it can be deduced that the inclusion of basalt and glass fibers in the matrix negatively impacts the composites’ dimensional stability, particularly at higher temperatures. Despite that fact, the dimensional changes beyond 80 °C occurred gradually and steadily.

## 4. Conclusions

This study was undertaken to investigate the mechanical, thermal, and chemical properties of hybrid BGRP composites. The FTIR data revealed that the incorporation of single and hybrid basalt and glass fibers in the matrix do not eloquently alter the composites’ chemical properties as no additional peaks were identified in the spectra. In future studies, chemical modification and grafting on the fiber prior to composite fabrication can be performed to promote chemical interactions between the components.

Moreover, the dynamic mechanical and thermal properties of hybrid BGRP composites were investigated using DMA. Among all the hybrid composites, only the B7.5/G22.5 composite portrayed a positive hybrid effect on the elasticity behavior, evidenced by a significantly high storage modulus value. It was deduced that an efficient stress transfer between the reinforcing fibers and the unsaturated polyester is achievable with a high proportion of glass fiber and a small amount of basalt fiber in the composite. The B7.5/G22.5 composite is also good in terms of viscous property, as revealed by the highest loss modulus value compared to the other hybrid BGRP composites. A higher glass fiber ratio yields remarkably better hybrid composite viscosity. Concerning the damping property, it appeared that all the hybrid BGRP composites showed no substantial improvement compared to the single reinforced GF composite. Despite having lower tan δ reductions than the single reinforced GF composite, the damping behavior of all the hybrid composites are still better than the neat matrix. The T_g_ values derived from the tan δ and loss modulus plots have revealed that the hybridization has marginally improved the thermal stability. The T_g_ had improved by up to 15 °C with glass and basalt fibers hybridization in a single matrix.

Lastly, the dimensional stability of the composites were evaluated using the thermomechanical analysis. Based on the result, it was found that the inclusion of basalt and glass fibers in the matrix negatively impacts the composites’ dimensional stability, particularly at higher temperatures. Therefore, the hybrid BGRP composite is deemed suitable for lower temperature applications.

Overall, the findings suggest that the hybridization of basalt and glass fibers in the unsaturated polyester formed composites with better mechanical and thermal properties than the single reinforced composites and a neat matrix.

## Figures and Tables

**Table 1:** Maximum mechanical properties of hybrid BGRP composites from previous studies.

No.	Resin	Hybrid	Fabrication Method	Tensile Strength (MPa)	Flexural Strength (MPa)	Impact Strength (kJ/m^2^)	Remark	Ref.
1	PP	Filler	Extrusion	31	-	-	Increasing basalt content led to a decrease in tensile strength.	[20]
2	Epoxy	Filler	Hand layup	273	497	426	The addition of a large amount of basalt reduced the impact strength.	[21]
3	Polyester	Laminate	Hand layup	78	175	-	Mechanical properties of BGRP composite are lower than BCRP laminates.	[22]
4	Epoxy	Laminate	Vacuum begging	448	-	-	The tensile strength of BGRP composite was lower than GFRP laminates.	[24]
5	Polyester	Laminate	Hand layup	293	302	192	The stacking sequence of BGRP improved mechanical properties.	[23]
6	Epoxy	Laminate	Vacuum begging	225	195	212	Increased glass fiber increased the tensile strength (BGRP < GFRP composite).	[25]
7	Epoxy	Laminate	Vacuum begging	-	-	4	Hybrid BGRP had a lower impact strength than the BFRP composite.	[26]
8	Polyester	Laminate	Hand layup	270	-	946	Hybrid BGRP offered the highest value of mechanical properties compared to GFRP and BFRP laminates.	[28]
9	Polyester	Laminate	Hand layup	246	-	204	Hybrid BGRP laminate had the highest tensile strength.	[29]

**Table 2:** Chemical constituents of basalt and E-glass fibers.

Chemical Components	Composition (wt%)	
	Basalt	E-Glass
SiO_2_ (silica)	57.5	55
Al_2_O_3_ (alumina)	16.9	15
Fe_2_O_3_ (ferric oxide)	9.5	0.3
MgO	3.7	3
Na_2_O	2.5	0.8
TiO_2_	1.1	-
K_2_O	0.8	0.2
B_2_O_3_	-	7
F	-	0.3

**Table 3:** Physical properties of basalt and E-glass fibers.

Properties	Basalt	E-Glass
Density	2.67	2.55–2.58
Modulus (GPa)	85–89	78–80
Strength (MPa)	2900–3100	2000–2500
Moisture (%)	0.008	0.1

**Table 4:** Basalt/glass fiber composition in hybrid composites.

No.	Composites	Sample Designation	Composition (wt%)		
			Matrix	Basalt	Glass
1	Five layers BF	BF	70	30	0
2	Four layers BF + two layers woven GF	B22.5/G7.5	70	22.5	7.5
3	Three layers woven GF + Three layers of BF	B15/G15	70	15	15
4	Two layers woven BF + three layers of GF	B7.5/G22.5	70	7.5	22.5
5	Five layers of woven GF	GF	70	0	30
6	UPE resin	UP	100	0	0

**Table 5:** Example calculation of hybrid composite (sample B15/G15).

Weight Percentage (wt%)	
Glass Fiber (wt%)	= 15 wt%
Basalt Fiber (wt%)	= 15 wt%
Unsaturated Polyester Resin (wt%)	= 70 wt%
MEKP (wt%) to UP resin	= 0.5% to UP resin
Density	
Glass Fiber	= 2.2 kg/L
Basalt Fiber	= 2.65 kg/L
Unsaturated Polyester Resin	= 1.87 kg/L
MEKP	= 1.152 kg/L
Volume	
Steel Mould	= 300 × 300 × 5 mm^3^ = 0.45 L
Glass Fiber	= 15% × 0.45 L = 0.0675 L
Basalt Fiber	= 15% × 0.45 L = 0.0675 L
Unsaturated Polyester Resin	= 70% × 0.45 L = 0.315 L
MEKP	= 0.5% × 0.315 L = 0.001575 L
Weight	
Glass Fiber	= 0.00675 L × 2.2 kg/L = 0.01485 kg = 148.5 g
Basalt Fiber	= 0.00675 L × 2.65 kg/L = 0.01788 kg = 178.8 g
Unsaturated Polyester Resin	= 0.315 L × 1.87 kg/L = 0.589 kg = 589 g
MEKP	= 0.001575 L × 1.152 kg/L = 0.0018144 kg = 1.8144 g

**Table 6:** Peak height and glass transition temperatures of hybrid basalt/glass reinforced unsaturated polyester resin composites.

Sample ID	Peak Height of Tan δ	Temperature (°C)	
		T_g_ from Tan δ	T_g_ from Loss Modulus
BF	0.49	81.37	71.48
B22.5/G7.5	0.52	84.47	70.80
B15/G15	0.46	83.36	69.85
B7.5/G22.5	0.48	80.24	69.70
GF	0.44	76.98	66.17
UP	0.71	80.24	55.72

## Data Availability

The data presented in this study are available on request from the corresponding author.
